# Exposure of Phosphatidylserine on *Leishmania amazonensis* Isolates Is Associated with Diffuse Cutaneous Leishmaniasis and Parasite Infectivity

**DOI:** 10.1371/journal.pone.0036595

**Published:** 2012-05-04

**Authors:** Jaqueline França-Costa, João Luiz Mendes Wanderley, Poliana Deolindo, Jessica B. Zarattini, Jackson Costa, Lynn Soong, Marcello André Barcinski, Aldina Barral, Valeria M. Borges

**Affiliations:** 1 Centro de Pesquisas Gonçalo Moniz/FIOCRUZ-BA, Salvador, Brasil; 2 Faculdade de Medicina, Universidade Federal da Bahia, Salvador, Brasil; 3 Pólo Universitário Macaé, UFRJ, Rio de Janeiro, Brasil; 4 Divisão de Medicina Experimental, Instituto Nacional do Câncer, Rio de Janeiro, Brasil; 5 Instituto Oswaldo Cruz, Rio de Janeiro, Brasil; 6 Instituto de Microbiologia Professor Paulo de Góes, Universidade Federal do Rio de Janeiro, Rio de Janeiro, Brasil; 7 Departments of Microbiology & Immunology and Pathology, the University of Texas Medical Branch, Galveston, Texas, United States of America; Royal Tropical Institute, The Netherlands

## Abstract

Diffuse cutaneous leishmaniasis (DCL) is a rare clinical manifestation of leishmaniasis, characterized by an inefficient parasite-specific cellular response and heavily parasitized macrophages. In Brazil, *Leishmania* (*Leishmania*) *amazonensis* is the main species involved in DCL cases. In the experimental model, recognition of phosphatidylserine (PS) molecules exposed on the surface of amastigotes forms of *L. amazonensis* inhibits the inflammatory response of infected macrophages as a strategy to evade the host immune surveillance. In this study, we examined whether PS exposure on *L*. *amazonensis* isolates from DCL patients operated as a parasite pathogenic factor and as a putative suppression mechanism of immune response during the infection. Peritoneal macrophages from F1 mice (BALB/c×C57BL/6) were infected with different *L*. *amazonensis* isolates from patients with localized cutaneous leishmaniasis (LCL) or DCL. DCL isolates showed higher PS exposure than their counterparts from LCL patients. In addition, PS exposure was positively correlated with clinical parameters of the human infection (number of lesions and time of disease) and with characteristics of the experimental infection (macrophage infection and anti-inflammatory cytokine induction). Furthermore, parasites isolated from DCL patients displayed an increased area in parasitophorous vacuoles (PV) when compared to those isolated from LCL patients. Thus, this study shows for the first time that a parasite factor (exposed PS) might be associated with parasite survival/persistence in macrophages and lesion exacerbation during the course of DCL, providing new insights regarding pathogenic mechanism in this rare chronic disease.

## Introduction

American cutaneous leishmaniasis is a disease caused by flagellated protozoa of the genus *Leishmania*. These organisms are obligatory intracellular parasites in mammalian hosts where they differentiate into amastigote forms, capable of proliferating inside macrophages and disseminating the disease [1]. Cutaneous leishmaniasis presents a wide spectrum of clinical manifestation in humans, varying from self-healing localized cutaneous leishmaniasis (LCL) to more severe forms such as mucocutaneous leishmaniasis (MCL) and diffuse cutaneous leishmaniasis (DCL) [2]. These different clinical forms depend mainly on the infecting *Leishmania* and host cell-mediated immune responses [3]. In Brazil, *L. amazonensis* infection can cause two distinct clinical forms of cutaneous leishmaniasis: LCL and DCL [2], [4]. While the clinical course of LCL is well characterized, the molecular mechanisms underlying DCL pathogenesis are still unclear. DCL is a rare clinical manifestation and is characterized by the appearance of several nonulcerated nodular skin lesions, uncontrolled parasite proliferation, resistance to most therapeutic strategies and absence or reduction of the cellular immune response against parasite antigens [2], [3]. Disseminated leishmaniasis (DL) is another rare clinical presentation of cutaneous leishmaniasis and might be confused with DCL since both have multiple lesions. However, the former is clinically characterized by the predominance of papules and acneiform type of lesions and high frequency of mucosal involvement. Additionally, DL patients show inhibition of the cell-mediated immune mechanisms, resulting in negative responses of the leishmanin skin test (LST) as well as the lymphocyte proliferation assay, but different from DCL, these patients have cellular immune responses totally restored with the conventional antimony therapy [5].

Phosphatidylserine (PS) is a phospholipid usually entrapped in the inner leaflet of the plasma membrane which, in some cases, is translocated to the outer cell surface [6]. During apoptotic cell death, exposed PS molecules become ligands for apoptotic cell recognition, leading to engulfment of the target cell and triggering the alternative activation of the phagocyte, characterized by elevated TGF-β_1_ and IL-10 production [7], [8]. Amastigote forms of *L. amazonensis* take advantage of exposed PS during the host cell infection inducing an anti-inflammatory response by macrophages and dendritic cells (DC). This creates a permissive environment for growth and dissemination [9] without necessarily progressing to apoptotic death in a mechanism called "apoptotic mimicry" [8], [10]. Pathogens such as *Toxoplasma gondii*, *Trypanosoma cruzi,* and vaccinia virus can also take advantage of PS recognition on their surface to induce an anti-inflammatory response and to inhibit microbicidal mechanism in order to establish infection in their respective hosts [11]–[13].

In addition, PS exposure on the surface of *Leishmania* promastigotes, as a consequence of apoptotic death due to nutrient deprivation, has been implicated as an important virulence factor in the establishment of infection by this parasite form. Van Zandbergen *et* *al*.[14] have showed that presence of apoptotic promastigotes of *L. major*, in an altruistic behavior, allows the intracellular survival of viable parasites. Alongside, our group showed that PS positive *L. amazonensis* promastigotes are present in the sand fly gut, being part of the infective inoculums in natural infections [15]. In this work, we examined whether PS exposure on *L*. *amazonensis* parasites, isolated from DCL lesions, is a possible mechanism of host immune suppression, thus playing a role in the pathogenesis of this chronic form of leishmaniasis.

## Materials and Methods

### Parasite isolates

Parasites were obtained by puncture and aspiration after previous asepsis and anesthesia of nodular lesions from DCL patients and of border ulcerated from LCL patients. Parasite strains were maintained in cryopreservation stock tanks since the time of isolation. *L.* (*L.*) *amazonensis* isolates from LCL (n = 5) or DCL (n = 7) patients were defrosted and cultivated in tubes with biphasic medium Novy-MacNeal-Nicolle (NNN), consisting of rabbit blood agar overlaid with Schneider's insect medium (Sigma Aldrich) pH 7.2 supplemented with 10% heat inactivated fetal bovine serum (FBS-Gibco) and 1% antibiotic (Gibco). Parasite isolates were expanded *in vitro* in complete Schneider medium, pH 7.2, at 25°C until they reached the stationary phase. Following expansion, aliquots of different strains of *L*. *amazonensis* were cryopreserved in complete RPMI medium containing 10% FBS (Gibco) and 1% DMSO until the moment of use and were not inoculated in animal during the study.

A total of 14 strains were used in this study (Table 1). All isolates used in these experiments were confirmed as *L*. *amazonensis* species by Multilocus Electrophoresis of Enzymes (MLEE) analysis as previously described [16]. This analysis was performed by *Leishmania* Collection of the Oswaldo Cruz Institute (CLIOC), FIOCRUZ, Rio de Janeiro, Brazil. The M2269 (MHOM/BR/1973/M2269) World Health Organization (WHO) reference strain was included in this study. As a control, we also used the LV79 (MPRO/BR/72/M 1841-LV-79) strain that was originally isolated from a case of LCL of primate, since its PS exposure mechanism have been well characterized [9].

**Table 1:** *Leishmania amazonensis* isolates used in this study.

Number	Strain	International Code (*)	Origin	Pathology
1	BA 106	MHOM/BR/1986/BA106	BA	DCL
2	BA 199	MHOM/BR/1989/BA199	MA	DCL
3	BA 276	MHOM/BR/1989/BA276	MA	DCL
4	BA 336	MHOM/BR/1990/BA336	MA	DCL
5	BA 700	MHOM/BR/1997/BA700	MA	DCL
6	BA 760	MHOM/BR/1999/BA760	MA	DCL
7	BA 820	MHOM/BR/2002/BA820	MA	DCL
8	BA 69	MHOM/BR/1985/BA 69	BA	LCL
9	BA 73	MHOM/BR/1985/BA 73	BA	LCL
10	BA115	MHOM/BR/1987/BA115	BA	LCL
11	BA125	MHOM/BR/1987/BA125	BA	LCL
12	BA113	MHOM/BR/1987/BA113	BA	LCL
13	M2269	MHOM/BR/1973/M2269	PA	LCL
14	LV79	MPRO/BR/72M 1841-LV-79	BR	LCL

(*) Code recommended for the *Leishmania* strain nomenclature, which includes the following data: host, country of origin, year when it was isolated, and original code (WHO, 1984).

### Epidemiological and clinical evaluations

Clinical and epidemiological characteristics of the patients with LCL and DCL are presented in Table 2. DCL patient's data were obtained between 1980 and 1990 [2] and were studied at the University of Bahia Hospital and in the Hospital dos Servidores do Estado do Maranhão, a state located in the Northeast of Brazil and were followed by Dr. Jackson Costa [2]. All DCL patients were diagnosed following previously described criteria [3], [17]. DCL patients presented prolonged natural history of their disease, negative response to leishmanin skin test (LST), intense number of parasitised macrophages, multiple nodular lesions all over the skin and chronic evolution of the disease, with several remissions. LCL patients were from State of Bahia, Brazil and were followed by Dr. Aldina Barral. Individuals with LCL presented a typical skin ulcer, positive skin test response to leishmanin (Montenegro test), duration of disease up to six months and presence of single or few ulcerated lesions [2].

**Table 2:** Clinical and immunologic data from patients with diffuse cutaneous leishmaniasis (DCL) and localized cutaneous leishmaniasis (LCL).

						Lesions		
Patients (N°)	Pathology	Sex	Age in the begining of the disease	Duration of disease (months)	LST	Nodules	Ulcers	Number
1	DCL	M	23	60	Neg.	++++	0	>500
2	DCL	M	06	144	Neg.	+++	6	51
3	DCL	M	20	180	Neg.	+++	0	168
4	DCL	M	04	276	Neg.	++	0	20
5	DCL	F	07	180	Neg.	+++	0	140
6	DCL	M	22	120	Neg.	+++	+	68
7	DCL	M	41	36	Neg.	++	0	22
8	LCL	M	64	04	+32mm	−	+	8
9	LCL	M	54	01	+25mm	−	+	2
10	LCL	M	70	02	+10mm	−	+	1
11	LCL	F	08	03	+12mm	−	+	5
12	LCL	M	27	06	+25mm	−	+	1

LST, Leishmanin Skin Test. –, no nodules; +, few; ++, moderate; +++, intense; ++++, very intense.

Written informed consent was obtained from all participants or legal guardians. All clinical investigations were conducted according to the principles expressed in the Declaration of Helsinki. The project was approved by the institutional review board from Centro de Pesquisas Gonçalo Moniz-FIOCRUZ/BA and Federal University of Maranhão.

### Mice

F1 (BALB/c x C57Bl/6) mice, age 6–8 weeks, were obtained from the animal facility of Research Coordination at the National Institute of Cancer (INCa- RJ, Brazil) or from Harlan Sprague Dawley (Indianapolis, IN, USA). This study was carried out in strict accordance with the recommendations in the Guide for the Care and Use of Laboratory Animals of the National Institutes of Health. All experimental procedures were approved and conducted according to the Brazilian Committee for Animal Experimentation (COBEA, Permit Number: L-036/08) and by Committee on the Ethics of Animal Experiments of the University of Texas Medical Branch, Galveston, TX (Permit Number: 9803016A). Surgery was performed under sodium pentobarbital anesthesia, and efforts were made to minimize mouse suffering.

### Macrophage infection

Thioglycollate-elicited peritoneal macrophages collected from F1 mice were plated and non-adherent cells were removed by washing in Hank's Balanced Salt Solution (HBSS-Sigma-Aldrich) after 2 h incubation at 37°C, 5% CO_2_. Promastigotes in stationary phase were added to adherent macrophages, at a 3∶1 ratio. After 2 h incubation at 34°C, free parasites were removed by extensive washing with PBS and cultures proceeded for an additional 5, 24 or 72 h post-infection. Bone marrow macrophages were generated as previously reported [18] and infected as described above. The cultures were fixed in 100% methanol and stained with Giemsa (Merck). The percentage of infected macrophages and the infectivity index (percentage of infected macrophages x average number of amastigotes per macrophage) were determined by randomly counting at least 200 macrophages per slide in light microscope, using the immersion objective (100X).

### Parasite quantification by real-time PCR analysis of parasite genomic DNA

As described in our previous report [19], parasite loads were quantified by measuring the amount of *L. amazonensis* cysteine proteinase isoform 1 (Llacys1) gene, which is a single-copy gene per haploid genome and is expressed in both developmental stages. Peritoneal macrophages from F1 mice were infected with stationary-phase promastigotes for 24 and 72 h and genomic DNA were extracted using a DNeasy kit (Qiagen). DNA (100 ng) was used for parasite detection at the University of Texas Medical Branch, the Real-time PCR Core Facility (all reagents were purchased from Applied Biosystems). Each sample was run in duplicate and normalized to the amount of total DNA extracted, and the number of parasites per sample was calculated based on a standard curve, as described in our previous studies [19].

### Parasitophorous vacuole (PV) morphometry

The sizes of the infected macrophages PVs induced by different isolates from patients with LCL and DCL were observed at Olympus microscopy and images were acquired using the software Image-Pro Plus 6.0 (Media Cybernetics). Values are shown as the area, in μm^2^, determined by width x length, for at least 60 PVs in each tested isolate.

### *In vitro* intracellular amastigote purification

Purification of intracellular amastigotes was performed using the protocol adapted from Wanderley et al. [9]. Briefly, 3×10^6^ thioglycolate induced peritoneal or bone marrow macrophages cultures were plated in 25-cm^2^ bottles and infected with stationary-phase promastigotes of *L. amazonensis*. After 24 h of infection, the cultures were washed with 5 ml of PBS and 3 ml of lysis buffer (20 mM Hepes, 0.25 M sucrose, 5 mM EDTA, 0.3 mM aprotinin, E-64 10 mM, Pepstatina 1 mM, pH 7.2) was added and incubated for 5 min. After this period, the macrophages were scraped from the bottle and lysed mechanically with a tissue grinder. The cell suspension was centrifuged at 50 g for 5 min at 4°C. The supernatant was carefully removed, further centrifuged, and washed two more times at 1450 g for 17 min at 4°C. Amastigotes were incubated under rotation for 2 h in DMEM (GIBCO) containing 4% FBS at 34°C to liberate the endocytic membranes [9], [20]. Parasites were centrifuged and washed 3 times with PBS and kept on ice until use.

### Flow cytometric analysis

The assessment of PS exposure was performed using the protocol adapted from Wanderley et al. [9]. In summary, 2×10^5^ amastigotes forms were washed, suspended in Annexin V binding buffer (ABB–10mM HEPES, 150 mM NaCl, 2.5 mM CaCl2) at pH 7.2. Cells were incubated at room temperature for 15 min with Annexin V-FITC (1/20 dilution; Molecular Probes) at the concentration indicated by the manufacturer. At the moment of acquisition, 0.4 µg/ml of propidium iodide (PI) was added to control and Annexin V-FITC-labeled samples. Data is shown as the difference between the geometric mean fluorescence intensity of unstained control samples and annexin V stained ones (ΔMFI). Data were collected in a BD FACScalibur and analyzed by Cellquest Pro (BD Biosciences). At least five thousand gated events were collected from each sample.

### Cytokine production

Macrophages were infected with different isolates of *L. amazonensis* in serum-free medium. Infected macrophages were treated with 100 ng/ml LPS from *Escherichia coli*, serotype 026:B6 (Sigma-Aldrich) for 20 h before collecting the supernatant for cytokines assay. After acidification to activate latent TGF-β_1_ followed by neutralization, total TGF-β_1_ was measured in the culture supernatants using ELISA according to the manufacturer's instructions (R&D Systems). Interleukin (IL)-10 and tumor necrosis factor-alpha (TNF-α) levels were measured using de Cytometric Bead Array Mouse Inflammatory kit (BD Biosciences) according to the manufacturer's protocol and analyzed by flow cytometry. The concentrations of the TGF-β_1_, IL-10 and TNF-α were determined by comparison with a curve generated from each cytokine standard, respectively.

### *In vivo* mouse infection

Six- to 8-wk-old wild-type and nude BALB/c mice were infected in the footpad with 2x10^6^ stationary-phase promastigotes of *L. amazonensis* (LV79 strain). PS exposure on purified amastigotes forms was evaluated weekly as previously described [20]. The animals were euthanized, and the footpad was removed under sterile conditions. The tissue was finely minced and homogenized with a tissue grinder. The cell suspension was ressuspended and centrifuged at 50 g for 10 min at 4°C. The supernatant was carefully removed, further centrifuged, and washed three more times at 1450 g for 17 min at 4°C. After 2-h incubation under rotation at 34°C to liberate endocytic membranes, the amastigotes were further centrifuged and incubated for 16 h at 34°C, at the end of which they were centrifuged and washed three times before use. Mice representative of both groups were sacrificed and histological analysis of infected tissues was made. The images were observed at Olympus microscopy and were acquired using the software Image-Pro Plus 6.0 (Media Cybernetics).

### Statistical analysis

Data are reported as median and interquartile interval of representative experiments and were analyzed using GraphPad Prism 5.0. Differences between groups were calculated using Kruskal-Wallis with Dunn's multiple comparison post test. Unpaired t test was used to compare differences regarding categorized variable. Mann-Whitney test was used to estimate significance in IL-10/TNF-α and TGF-β/TNF-α ratios from LCL or LCD macrophage infection. Spearman test was used to verify the significance in the correlation tests. Each experiment was repeated at least three times. Differences were considered significant at *p*<0.05.

## Results

### *Leishmania amazonensis* amastigotes from DCL and LCL patients expose different amounts of PS on their surface

**Figure 1:**
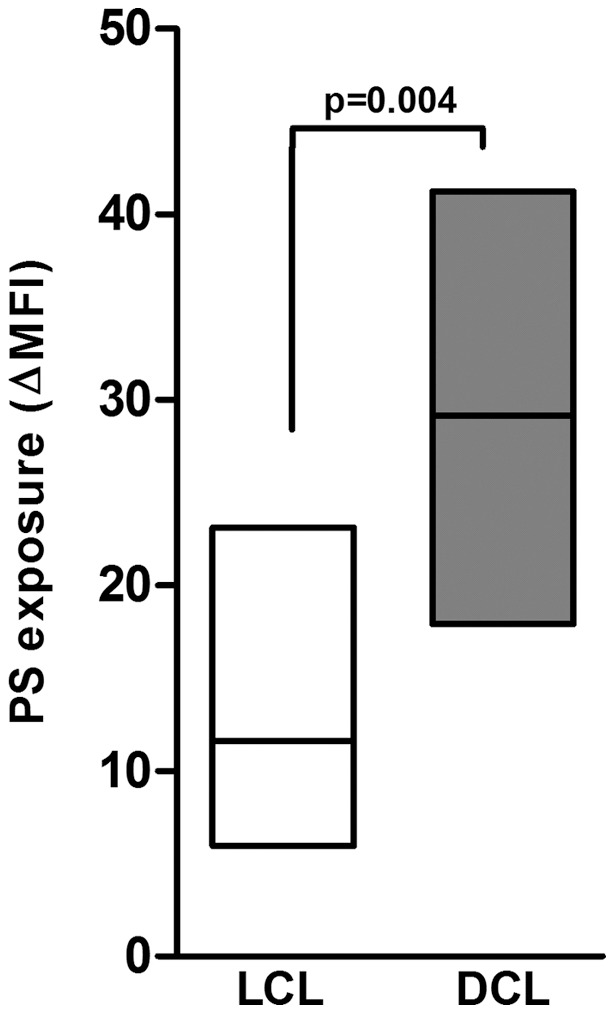
PS exposure on the *L. amazonensis* amastigotes surface. Thioglycollate-induced peritoneal macrophages derived from F1 (BALB/C X C57BL/6) mice were infected with different isolates obtained from patients with LCL (BA69, BA73, BA115, BA 125, and M2269) (□ ) and DCL (BA106, BA276, BA336, BA700, and BA760) (▪ ) at a 3∶1 parasite-to-cell ratio. After 24 h of infection, amastigotes were purified for PS exposure analysis by flow-cytometry, as described in Methods. One representative experiment of at least five independent repeats is shown. Boxes represent median values and interquartile interval from different isolates mentioned above. Differences were checked using Unpaired t test.

Our group has shown that PS exposure on the external layer of the amastigotes cell membrane is modulated by the host, being higher on parasites recovered from BALB/c mice, compared with parasites derived from C57BL/6 mice [9]. Based on these data, we used F1 (BALB/c x C57BL6) mice to avoid the interference of the host genetic background that could influence a possible modulation of PS exposure. To check the amount of PS on *L. amazonensis* amastigotes derived from DCL and LCL isolates, thioglycolate-induced peritoneal macrophages from F1 mice were infected *in vitro* with stationary-phase promastigotes. After macrophage disruption, purified amastigotes were stained with Annexin V and PI. We considered the gate annexin V^+^/PI^-^ for PS exposure analysis. As shown in Figure 1, at 24 h after infection, DCL-derived amastigotes (BA106, BA276, BA336, BA700, and BA760) presented higher levels of PS exposure (median ± SD, ΔMFI of 28.9±3.9) than the LCL-derived parasites (BA69, BA73, BA115, BA 125, and M2269) (ΔMFI of 10.7±2.6) (*p* = 0.01). The difference between the groups was no longer observed at 48 h and remained unchanged until 72 h post-infection (data not shown). Moreover, LV79, a long-term maintained *L. amazonensis* strain in laboratory, showed PS exposure comparable to LCL isolates. Independent assays were performed with all isolates included in this study (table 1) and the same profile of PS exposure was observed. Similar results were observed using bone marrow-derived macrophages (data not shown). Therefore, the high levels of PS exposure in parasite isolates from DCL patient group were not specific to the source of macrophages.

### Differential PS exposure modulates the infectivity of LCL and DCL isolates

**Figure 2:**
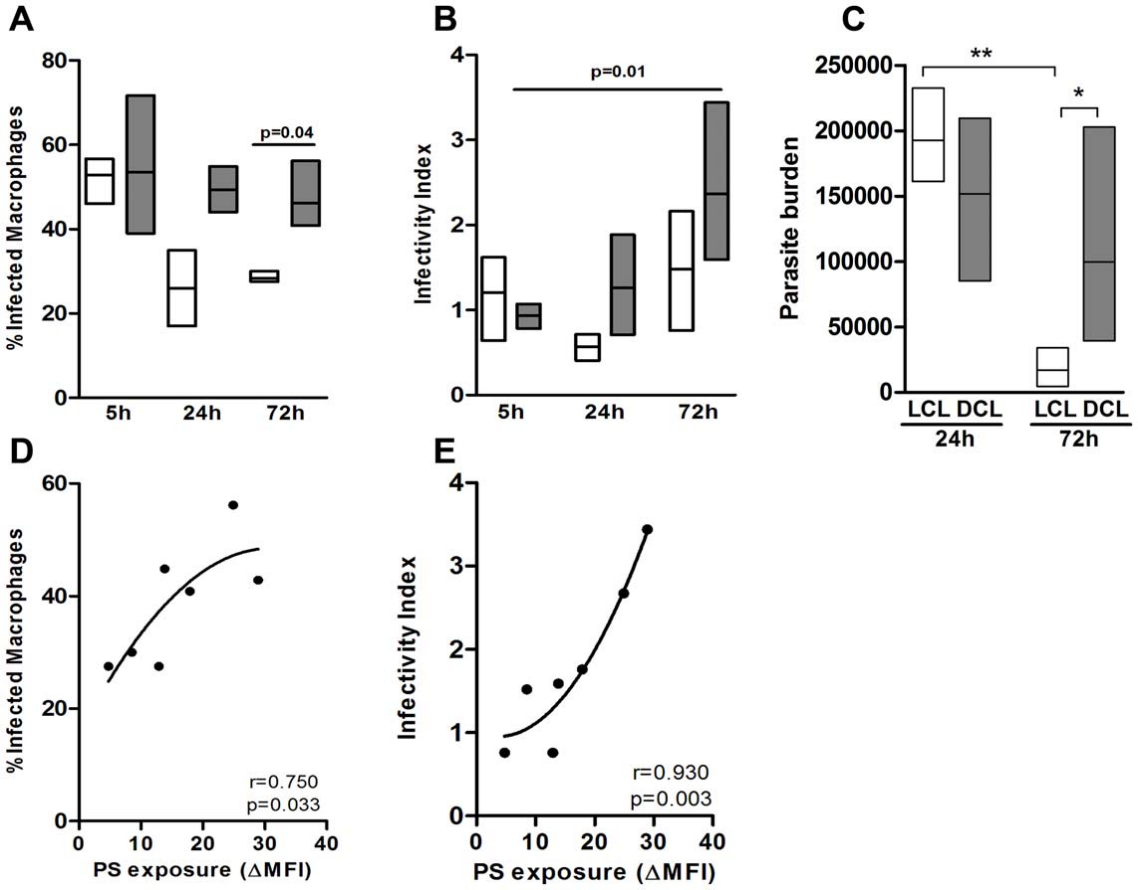
*Leishmania* isolate infectivity and PS exposure. Peritoneal macrophages derived from F1 mice were infected with different isolates obtained from patients with LCL (BA69, BA73, BA 125, and M2269) (□) and DCL (BA276, BA336, and BA700) (▪). After 5, 24 and 72 h of infection, cells were fixed and stained. The percentage of infected macrophages (**A**) and infectivity index (**B**) were defined under a microscope. Parasite burden was measured by quantitative PCR at 24 and 72 h (**C**). Boxes represent median values and interquartile interval from different isolates mentioned above. The correlations between PS exposure at 24 h with percentage of infected macrophages and infectivity index in 72 h are showed in (**D**) and (**E**), respectively. The four lower points in X axis (PS exposure) represents LCL isolates while the three higher points are from DCL isolates. One representative experiment of at least three independent repeats is shown. Kruskal-Wallis was used with Dunn's Multiple Comparison post-test. Spearman test was used to verify the correlations. The r values are plotted in each graph.

To rule out differences in metacyclogenesis we characterized the percentage of metacyclics promastigotes in *L. amazonensis* isolates from DCL and LCL patients as described by Saraiva et al. [21]. There were no differences regarding metacyclogenesis between both groups (data not shown). To evaluate the infection profile of the different LCL and DCL *L. amazonensis* isolates, we infected F1 peritoneal macrophages with stationary-phase promastigotes at a 3∶1 parasite-to-cell ratio. The percentage of infected macrophages (Figure 2A) and the infectivity index (Figure 2B) were similar at 5 h post-infection for both groups, indicating comparable rates of internalization for both DCL (BA276, BA336, and BA700) and LCL (BA69, BA73, BA 125, and M2269) parasites. However, the percentage of infected macrophages was significantly higher at 72 h post-infection with DCL isolates (49%±6.8) when compared to LCL isolates (26%±1.4, *p* = 0.04). In addition, the infectivity index of DCL parasites increased more than 2-fold, from 5 h (0.94 ± 0.13) to 72 h post-infection (2.21±0.85, *p* = 0.01), while the infectivity index of the LCL isolates was relatively constant, indicating that proliferation of DCL isolates was more intense. Quantitative PCR analysis of parasite loads at 24 and 72 h confirmed that DCL isolates proliferated more efficiently than LCL isolates (Figure 2C), corroborating results obtained by optical microscopy (Figure 2B). To check whether differential PS exposure by amastigotes observed at 24 h post-infection was related with difference in infectivity among the isolates at 72 h post-infection, we applied correlation statistics tests between these variables. There was a positive correlation between PS exposure and the percentage of infected macrophages (Figure 2D, r = 0.756, *p* = 0.033) and infectivity index (Figure 2E, r = 0.942, *p* = 0.008), suggesting that the differential PS exposure at 24 h might affect the parasite load at 72 h.

### Profile of cytokine production

**Figure 3:**
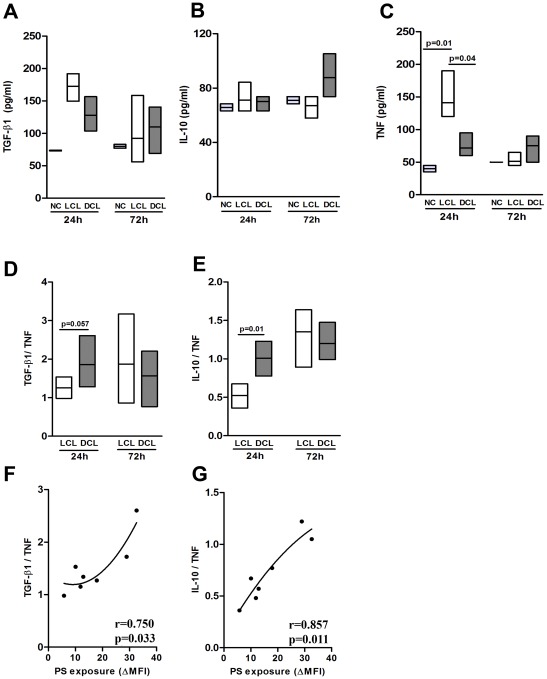
Cytokine production by infected macrophages with different *Leishmania amazonensis* isolates. Peritoneal macrophages of F1 mice were infected with isolates obtained from LCL (BA69, BA73, BA 125, and M2269) (□) and DCL (BA276, BA336, and BA700) patients in a serum-free medium containing 100 ng/ml LPS. Negative control (NC) represents uninfected macrophages. Supernatants were collected after 24 and 72 h and active TGF-β_1_ (**A**) production was assayed by ELISA. IL- 10 (**B**) and TNF-α (**C**) production were assessed by Cytometric Bead Array (CBA). The TGF-β/TNF-α (**D**) and IL-10/TNF-α (**E**) ratios. Boxes represent median values and interquartile interval of the ratios from different isolates mentioned above. The correlations between PS exposure at 24 h with TGF-β/TNF-α and IL-10/TNF-α at 24 h are showed in (**F**) and (**G**), respectively. The four lower points in X axis (PS exposure) represents LCL isolates while the three higher points are from DCL isolates. Differences were checked using Kruskal-Wallis with Dunn's multiple comparison post test. Spearman test was used to verify the significance in the correlations between cytokine ratio and PS exposure. The r values are plotted in each correlation graph.

To assess whether the infection with DCL or LCL *L. amazonensis* isolates distinctly modulates the inflammatory activity of host cells, we evaluated the levels of TGF-β_1_, TNF-α and IL-10 in the supernatants of peritoneal macrophages infected with either DCL (BA276, BA336, and BA700) or LCL (BA69, BA73, BA 125, and M2269) isolates. We find significant difference between the two groups regarding cytokine production for TNF-α at 24 h post-infection (Figure 3C), but not for TGF-β1 (Figure 3A) and IL-10 (Figure 3B). Moreover, the ratio of TGF-β1/TNF-α (Figure 3D) and of IL-10/TNF-α (Figure 3E) production were higher in DCL than LCL isolates at 24 h post-infection. In fact, we found that PS exposure on DCL amastigotes at 24 h post-infection displayed a positive correlation with the TGF-β_1_/TNF-α ratio (Figure 3F, r = 0.75, *p* = 0.03) and with the IL-10/TNF-α ratio (Figure 3G, r = 0.88, *p* = 0.01), suggesting that the anti-inflammatory phenotype induced by macrophages infected with DCL isolates is linked to PS exposure on the parasite surface.

### PS exposure on the parasite surface is associated to PV size

**Figure 4:**
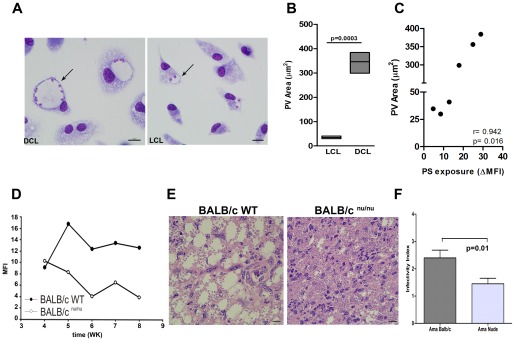
Parasitophorous vacuole analysis. Photomicrographs of macrophages from F_1_ mice, infected with DCL (BA276) and LCL (BA125) isolates after 72 h of culture (**A**). Arrows point to individual PVs. Magnification 400X. The average sizes of the PVs induced by different isolates from patients with LCL (BA69, BA73 and BA 125) (□) and DCL (BA276, BA336, and BA700) (▪) after 72 h of infection were measured using Image-Pro Plus 6.0 (**B**). Data are shown as the area in μm^2^ of PVs in each tested isolate. Boxes represent median values and interquartile interval of PV sizes pooled from different isolates mentioned above. The correlation between PS exposure at 24 h and PV area showed in (**C**). The three lower points in X axis (PS exposure) represents LCL isolates while the three higher points are from DCL isolates. Differences were checked using unpaired t test. Spearman test was used to verify the significance in the correlations between PV area and PS exposure. The r value is plotted in the correlation graph. PS exposure on the *L. amazonensis* amastigotes surface from nude mice (**Figures** **D–F**). Mean fluorescence intensity of annexin V staining on lesion-derived amastigotes (LV79) purified from BALB/c WT or BALB/c ^nu/nu^ mice (**D**). Graph corresponds to one representative experiment out of three. H&E staining of histological slides of infected footpads obtained from BALB/c WT or BALB/c nude mice 5 weeks post infection (**E**). Peritoneal macrophages derived from BALB/c mice were infected with lesion-derived amastigotes (LV79) purified from BALB/c WT or BALB/c ^nu/nu^ mice 5 weeks post infection. After 24 h of infection cells were fixed and stained. The infectivity index (**F**) was defined by microscopic analysis. Representative experiment of two repeats. Differences were checked using unpaired t test.

One of unique features of *L. amazonensis* infection is the induction of large parasitophorous vacuoles (PVs) within infected macrophages [22], [23]. The formation of such organelles is linked to the capacity of the parasite to resist against macrophage activation [24]. As shown in figure 4A, another marked difference between DCL and LCL infection was the PV size. While DCL infection induced large PVs, LCL infection showed tight vacuoles at 72 h. There was no difference between groups relative to size of the PVs at 4 and 24 h (data not shown). In order to quantify the PV sizes induced by DCL (BA276, BA336, BA700) and LCL (BA69, BA73, BA 125) isolates at 72 h of infection (Figure 4B), the PV size of Giemsa-stained macrophages was determined by width x length and presented as an averaged area (in µm^2^). As shown in Figure 4B, PV sizes induced by DCL isolates (346.4±43.47) were significantly higher than those induced by LCL isolates (35.12±5.48, *p* = 0.0003). Furthermore, there was a highly positive correlation between PV size and PS exposure on the parasite surface. The DCL isolates displaying higher PS exposure at 24h of infection, were also those inducing the largest vacuole sizes (Figure 4C, r = 0.942, *p* = 0.016).

Based on evidences that PS exposure in amastigotes is induced by the host immune response (Wanderley et al. manuscript in preparation), we compared the intensity of PS exposure in *L. amazonensis* (LV79) amastigotes obtained from Balb/c^nu/nu^ and wild-type mice. Indeed, at 5–8 weeks post-infection, amastigotes obtained from nude mice exposed 2–4 fold less PS at their surface than did parasites obtained from wild-type counterparts (Figure 4D). To characterize the role of PS exposure on PV formation, we performed histological analysis of footpad lesions of wild-type and nude mice. At 5 weeks post-infection, the majority of infected macrophages observed in wild-type mice lesions presented the characteristic large PVs; however, we could not detect the occurrence of large PVs in nude mice, regardless of the presence of a large number of parasites (Figure 4E). Moreover, lesion-derived amastigotes purified from immune deficient mice (at 5 weeks post-infection) presented reduced infectivity index compared with parasites derived from wild-type mice (Figure 4F).

### PS exposure on *L. amazonensis* isolates correlates with clinical disease

**Figure 5:**
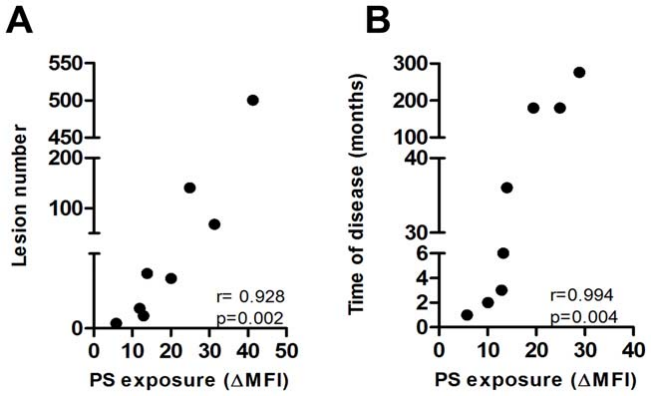
PS exposure on *L. amazonensis* isolates correlates with clinical parameters of the disease. Correlation between clinical parameters and PS exposure in isolates from DCL (BA276, BA336, BA700 and BA760) and LCL (BA69, BA73, BA 115, and BA125) at 24 h post-infection. The four lower points in X axis (PS exposure) represents LCL isolates while the four higher points are from DCL isolates. Spearman test was used to verify the significance in the correlations between PS exposure in 24 h with lesions number (**A**) and time of disease (**B**). The r values are plotted in each correlation graph.

To check whether the differences in PS exposure found in isolates from DCL (BA276, BA336, BA700 and BA760) and LCL (BA69, BA73, BA 115, and BA125) patients could be associated with different clinical parameters (Table 2), we performed correlation statistics between these variables, using parasite PS exposure at 24 h post-infection and clinical data, respectively. These results showed that isolates which presented a higher PS exposure on their surface, were derived from DCL patients with the higher number of lesions (Figure 5A, r = 0.928, *p* = 0.002) and duration of the disease (Figure 5B, r = 0.994, *p* = 0.004). These data suggest that, in human DCL, parasites that are capable of exposing higher amounts of PS induce a more severe and persistent disease.

## Discussion

The factors that determine DCL disease outcome remain poorly understood and may be associated with immunological and genetic features of the patients, as well as with pathogenic factors of the parasite [2], [3], [25], [26]. *L. amazonensis* is the main etiologic agent of DCL in South America [4], although it can be associated with the entire spectrum of American cutaneous leishmaniasis [2], [27]. In the experimental models, *L. amazonensis* infection can cause non-healing lesions in nearly all tested mouse strains, via various mechanisms such as inhibition of antigen-presenting cell functions, compromising the activation of effector lymphocytes and exposure of PS on their surface in attempt to evade the defense mechanisms of host cells [9], [18], [28]-[30].

Here we examined whether PS exposed on the surface of *L. amazonensis* amastigotes obtained from DCL patients can operate as a parasite pathogenic factor, repressing the host immune response. We show, for the first time that, PS exposure by amastigotes is associated with a modified host inflammatory response, correlating with parasite infectivity and with clinical parameters of DCL. We used *L*. *amazonensis* stocks isolated from DCL or LCL patients to infect murine macrophages and assessed PS exposure on the surface of purified intracellular amastigotes. The observation of increased PS exposure in isolates from DCL patients was consistently reproduced. The maintenance of the capacity to expose PS indicates that this is a relatively stable phenotype, controlled by unknown mechanisms. We showed that parasites isolated from patients with DCL are able to expose more PS moieties, in the first 24 h of interaction with murine macrophages, when compared to amastigotes from LCL patients. This difference on PS exposure correlates with parasite infectivity. Indeed, those exposing high levels of PS on the surface, the DCL isolates, had the higher percentage of infected cells at 72 h and the ability to proliferate inside macrophages throughout the course of infection. In this context, PS exposure in DCL isolates seemed to be associated with parasite survival/persistence within the host cell. The host cells used for the *in vitro* infection were (BALB/c x C57BL/6) F1 macrophages, because PS exposure on amastigotes is modulated depending on the host background [9]. This mice strain displays a similar pattern of lesion development when compared to C57BL/6 mice, with establishment of chronic lesions, less severe than the ones observed in BABL/c mice [9]. Little is known about the inheritance patterns of resistance/susceptibility loci, particularly for *L. amazonensis* infection. In other models, the genetic predisposition for susceptibility or resistance in mice correlates with gene loci that control the dominance of Th2 or Th1 responses, respectively [31]. Nevertheless this dichotomy does not apply to the experimental models of *L. amazonensis* infection.

Given that intense immune suppression is a hallmark of DCL patients [32], it is conceivable that parasites with increased PS exposure can contribute to disease suppression. The PS exposure on *Leishmania* surface has been previously described by our group as a strategy to infect and avoid host immunity, as well as for other intracellular pathogens such as *Trypanosoma cruzi* [12] and *Toxoplasma gondii* [11]. The results with *L. amazonensis* isolated from DCL patients reinforce the PS recognition as a central event inducing macrophage deactivation.

It is important to emphasize that parasite-associated factors, other than PS, may also contribute to parasite survival/persistence within the macrophage and to lesion exacerbation during the course of human leishmaniasis. In this regard, *L. amazonensis* isolated from patients with different forms of leishmaniasis showed differences in resistance to NO, and patients infected with NO-resistant isolates had significantly larger lesions than those infected with NO-susceptible isolates [33]. Although this issue was not addressed here, we cannot rule out a distinct resistance to NO between isolates from DCL and LCL patients as a possible explanation for the difference in parasite burden in infected macrophages.

DCL patients with active disease show increased IL-10 expression in PBMCs [32]. Indeed, we observed that *L. amazonensis* isolates from DCL patients preferentially induced an anti-inflammatory profile of cytokines with a significant increase in the IL-10/TNF-α ratio and were more competent to infect and proliferate inside macrophages. These events were clearly correlated with PS exposure on parasites. Several groups demonstrated that *L. amazonensis* is able to deactivate intracellular pathways that lead to innate immune cell activation, such as degradation or decreased phosphorylation of STAT 1, 2, 3 and ERK1/2, reduced expression of interferon regulatory factors 1 and 8, and activates a classic transcriptional repressor, the p50/p50 NF-KB complex, thereby reducing mRNA levels of iNOS [15], [30]. In addition, the expression of activation markers, MHC class II, cytokines and chemokines is abrogated during the *L. amazonensis* infection [29], [34]. Nevertheless, it is important to notice that these mechanisms were described for *L. amazonensis* strains maintained in laboratory. Here we used parasites isolated from DCL and LCL patients that might trigger different signaling pathways in parasite-host cell interplay, but this hypothesis needs to be further investigated.

A striking feature of the histological analysis of biopsies performed in DCL patients is the presence of largely vacuolated macrophages [2]. Interestingly, our *in vitro* results with DCL isolates reproduced what is observed in patients. It was previously demonstrated that large vacuoles formed by *L. amazonensis* amastigotes are the result of macropinocytosis induced by the parasite and are dependent on PS exposure [9]. Here, we showed that differential PS exposure on parasites isolated from patients with either DCL or LCL is associated with PV sizes. Although our data suggest the involvement of PS molecule in the large vacuole induction during L. amazonensis infection, we cannot discard the possibility that other mechanisms are involved. The increased expression of LYST/Beig, a gene related to the size of lysosomes [35], and molecules secreted by the parasite into the vacuole [36] may also contribute to the formation of vacuoles characteristic of L. amazonensis infection. Albeit the mechanisms by which *L. amazonensis* species manipulate the formation of large PVs are still unclear, it has been suggested that PV expansion may protect *L. amazonensis* from host microbicidal pathways, by diluting the proteolytic enzymes present in the PV [24] and favoring parasite replication within host cells [35]. Thus, it is possible that multiple mechanisms may contribute to the formation/maintenance of large PVs with heavy parasite loads in DCL lesions.

Previous studies from our group showed that PS exposure by amastigotes is regulated by the host cells [9]. However, how the host immune system participates in the modulation of parasite PS exposure is not clear yet. Here we observed that parasites purified from wild-type BALB/c mice footpad lesions exposed higher amounts of PS than those purified from T cell-deficient BALB/c^nu/nu^ mice. In addition, large vacuoles are present in wild-type but not in nude mice, even though footpad lesions of immune-deficient mice contained appreciable amounts of parasites. Moreover, lesion-derived amastigotes purified from immune-deficient mice presented reduced infectivity index compared with wild-type mice strengthening the role of PS exposure for survival and proliferation of intracellular parasites. Although these results are not directly linked to DCL and LCL isolates, they reinforce the importance of PS exposure in PV induction. On the other side, our findings of differences in the PV areas suggest an association, in a clinical setting, between enlarged PV size and immunodeficiency. Actually, PS exposure on intracellular amastigotes seems to be modulated by interactions between infected macrophages and CD4^+^ T cells (Wanderley et al., in preparation). In this regard, it is possible that the low amounts of PS on amastigotes surface displayed from nude mice are due to deficient T cell activation.

The diagnosis of DCL disease was validated by a set of clinical, immunological, and histological parameters showing large PV heavily parasitized. DCL patients had negative response to leishmanin skin test, indicating the absence or low cell-mediated response to parasite antigens, numerous lesions and a long period of illness. Additionally, these patients also had numerous relapses during treatment (Table 2). The greater number of lesions and a longer duration of disease in DCL patients were associated with increased PS expression in parasites isolated from these patients. Therefore, it is also possible that recognition of PS by the host cells increases macrophage permissiveness to parasite growth and contributes to the maintenance of infection.

Despite that the PS exposure mechanism in *L. amazonensis* amastigotes seems to be under the control of host adaptive immune responses, whether or not these parasites acquire PS from the host or from endogenous sources is still an open question. Several lines of evidence strongly point to the active PS exposure and rescue from apoptotic death as the most probable explanation for the presence of PS^+^ amastigotes. Our data suggest that increased PS exposure on *L. amazonensis* isolated from patients with DCL is an important and novel mechanism for parasite survival, dissemination and disease outcome. However it is necessary to gain insight into the mechanisms by which parasite PS exposure is modulated in DCL patients. This understanding will be important to extend our knowledge about the immunopathogenesis of DCL disease and will open new perspectives for therapeutic interventions.
